# Dose Optimization of Cefazolin for Pediatric Patients with Renal Impairment Using Physiologically Based Pharmacokinetic Modeling (PBPK) in GastroPlus

**DOI:** 10.3390/pharmaceutics18101260

**Published:** 2026-10-05

**Authors:** Abdelrahman Abdeen, Yousef A. Bin Jardan, Najia Rahim, Ruqaia Alshami, Raimar Löbenberg, Muhammad Sarfraz

**Affiliations:** 1 College of Pharmacy, Al Ain University, Al Ain P.O. Box 64141, United Arab Emirates; 2 Department of Pharmaceutics, College of Pharmacy, King Saud University, Riyadh 11451, Saudi Arabia; 3 Department of Pharmacy Practice, Dow College of Pharmacy, Dow University of Health Sciences, Karachi 74200, Pakistan; 4 3142K Katz Group Centre for Research, Faculty of Pharmacy and Pharmaceutical Sciences, University of Alberta, Edmonton, AB T6G 2H5, Canada

**Keywords:** PBPK, transporters, pediatrics, fold errors, model development

## Abstract

**Background/Objectives**: Cefazolin is a first-generation cephalosporin antibiotic widely used for the treatment and prophylaxis of bacterial infections in both adult and pediatric populations. It is predominantly eliminated unchanged by the kidneys. Impaired renal function can reduce its clearance and increase systemic exposure. This study aimed to develop and validate a PBPK model of cefazolin using GastroPlus™ 9.9 to optimize dosing in pediatric patients with renal impairment. **Methods**: A cefazolin PBPK model incorporating renal transport processes was developed using clinical data from healthy adults, subsequently evaluated in adults with renal impairment, and scaled to healthy pediatrics following validation in these populations. The model was extrapolated to pediatric patients with varying degrees of renal impairment. Dose optimization was performed based on predicted exposure, followed by pharmacodynamic evaluation using unbound fractions and a selected MIC of 4 µg/mL. **Results**: The model demonstrated good agreement between observed and predicted pharmacokinetic parameters including C_max_ and AUC_0-∞_ across the evaluated populations. Dose reductions of approximately 43%, 80%, and 90% relative to the standard pediatric dose were identified in moderate RI, severe RI, and ESRD, respectively, together with interval extension. Predicted %fT > MIC was 55.5%, 58%, 83%, and 81% for mild RI, moderate RI, severe RI, and ESRD, respectively. For surgical prophylaxis, unbound cefazolin concentrations remained above the MIC for 4.5–38.8 h across the RI groups, supporting maintenance above the MIC during a simulated 4 h surgical period. **Conclusions**: The PBPK model adequately describes cefazolin pharmacokinetic in healthy adults, adults with CKD, and healthy pediatric subjects and provides a model-informed basis for dose and interval adjustment in pediatric RI. The proposed regimens are model-based extrapolations requiring clinical confirmation, especially because the pediatric RI model was not independently validated against observed pediatric RI data.

## 1. Introduction

Cefazolin, a semi-synthetic first-generation cephalosporin, is the most commonly used agent for surgical antimicrobial prophylaxis (SAP) in hospitals. It provides good activity against Gram-positive bacteria, including most streptococci and staphylococci, particularly for those with methicillin-sensitive *Staphylococcus aureus* (MSSA). This choice is based on several factors, including its coverage, safety profile, efficacy and low cost [1,2]. In adults, cefazolin is administered by intravenous (IV) or intramuscular (IM) injection. Adult dosing ranges from 1 to 2 g every 8 h depending on the type of infection. However, for surgical prophylaxis, it is administered as 2 g or 3 g if the patient’s weight is 120 kg or more, as a 30–60 min infusion prior to surgery, with the possibility of re-dosing after 4 h [3].

Cefazolin is predominantly eliminated through the kidneys, with up to 80% of the administered drug excreted in urine [4]. Its elimination half-life is approximately 1.8 h in adults and 1.95 h in children, and it is prolonged in patients with renal impairment [5]. Therefore, renal function is an important determinant of cefazolin elimination and systemic exposure.

Cefazolin dosage needs to be adjusted in people with renal impairment (RI) since renal function significantly affects its elimination. To avoid drug accumulation while maintaining adequate antimicrobial exposure, clinical recommendations typically adjust the dosage schedule based on the degree of renal impairment by either reducing the dose or extending the dosing interval [6,7]. While more severe renal failure may necessitate both dose reduction and interval extension, moderate renal impairment may allow the dosing interval to be extended while retaining the standard dose [7,8]. These modifications aim to minimize excessive accumulation and the risk of toxicity while maintaining appropriate antibacterial exposure. Recommendations derived from clinical data at the population level, however, might not adequately consider interindividual differences in renal function and other physiological parameters.

According to the 2013 recommendations of the Surgical Infection Society (SIS), the American Society of Health-System Pharmacists (ASHP), and the Infectious Diseases Society of America (IDSA), cefazolin should be administered for pediatric surgical prophylaxis at a weight-based dose of 30 mg/kg; however, the guidelines also acknowledge that this dosing recommendation is not strongly supported by pediatric pharmacokinetic evidence [3]. In children with renal impairment, dose adjustment is generally based on the degree of renal dysfunction and may involve reducing the dose, extending the dosing interval, or applying both approaches for reduced renal clearance [9,10]. However, pediatric renal dosing recommendations are supported by limited pharmacokinetic evidence and are frequently extrapolated from adult data, with relatively few recommendations based on dedicated pediatric clinical studies [9]. Population pharmacokinetic studies can provide additional information on cefazolin disposition and variability in children, but their use for establishing renal dose adjustment regimens remains limited by the scarcity of pediatric patients with renal impairment. Consequently, current clinical dosing approaches may not fully account for interindividual variability in renal function and drug disposition, highlighting the need for mechanistic approaches to support pediatric cefazolin dose optimization in renal impairment.

Because antibacterial activity is linked to the amount of time that the drug concentration stays above the minimum inhibitory concentration (MIC), typically expressed as the percentage of the dosing interval during which concentrations exceed the MIC (%T > MIC), adequate exposure to cefazolin is necessary to achieve its pharmacodynamic effect. Therefore, whereas insufficient adjustment may lead to excessive accumulation, excessive dose reduction or extension of the dosing interval in patients with renal impairment may reduce the duration of exposure above the MIC. The Clinical and Laboratory Standards Institute (CLSI) revised the cefazolin susceptibility breakpoint for Enetrobacterales to ≤2 µg/mL in 2011, based on a 2 g every 8 h dosing regimen and supporting pharmacokinetic/pharmacodynamic target attainment data [11].

Developmental changes in body size, organ function, and renal maturation can affect drug clearance and systemic exposure throughout childhood, complicating the disposition of cefazolin in children [12,13]. Furthermore, despite the physiological distinctions between adults and children, pediatric renal dosage recommendations are often extrapolated from adult data. Only 14% of the recommendations in the Lexicomp Pediatric and Neonatal Dosage Handbook were backed by a pediatric clinical study, according to an analysis of pediatric renal dosage guidelines [9]. Therefore, developing evidence-based cefazolin dosing regimens is difficult due to the scarcity of pediatric pharmacokinetic data, especially in children with RI.

A mechanistic method for combining drug-specific properties with physiological changes associated with aging and diseases is physiologically based pharmacokinetic (PBPK) modeling. Renal impairment can be modeled by disease-related changes in renal function and drug clearance, while developmental changes in body size, organ function, blood flow, and renal function can be incorporated into PBPK models in pediatric populations [14,15,16]. When clinical pharmacokinetic data are scarce, this method can improve dose optimization and help estimate drug exposure across pediatric groups.

A mechanistically based strategy is still required to optimize cefazolin dosing in pediatric patients with variable degrees of renal impairment (RI), even with the established clinical usage of cefazolin and the availability of renal dose adjustment recommendations for adults. In order to provide appropriate antimicrobial exposure while lowering the risk of drug accumulation and toxicity, the aim of this study was to develop and validate a cefazolin PBPK model using GastroPlus^®^ and to optimize cefazolin dosing regimens in pediatric patients across various stages of RI.

## 2. Materials and Methods

### 2.1. Modeling Software

GastroPlus^TM^ 9.9 (Simulations Plus, Inc., Lancaster, CA, USA) was used to develop the PBPK model of cefazolin in both healthy and renally impaired populations. Populations were built using Population Estimates for Age-Related (PEAR^TM^) physiology within GastroPlus^®^ (version 9.9). Plasma drug concentration profiles were obtained from clinical trials using Digit 1.0.4. (Simulations Plus, Inc., Lancaster, CA, USA). Those profiles were evaluated using PKPlus Module (version 2.5) in GastroPlus^®^ 9.9.

### 2.2. Cefazolin PBPK Model Development

**Figure 1:**
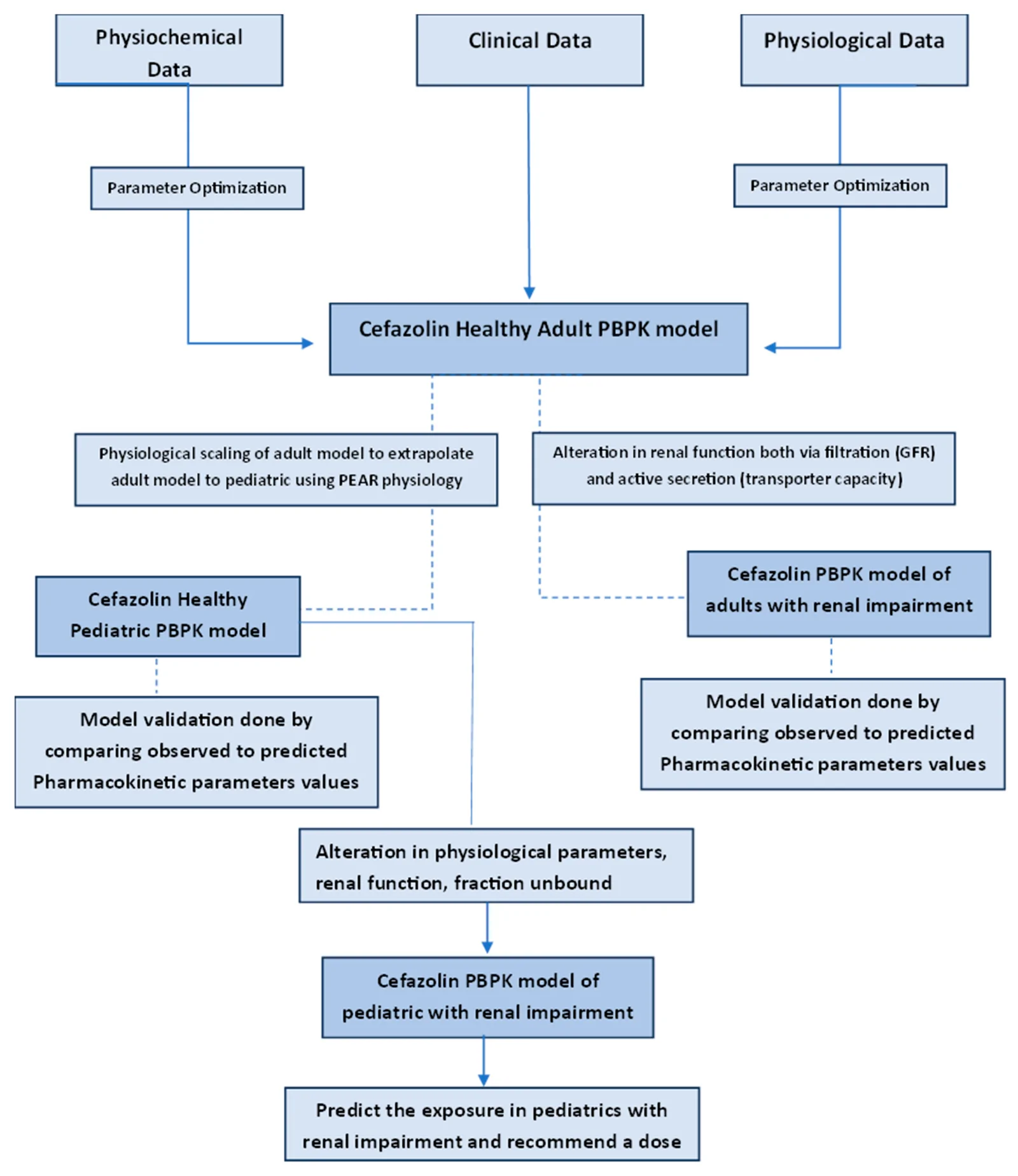
Schematic workflow adopted for physiological based pharmacokinetic (PBPK) model of cefazolin.

To develop the PBPK model, we integrated the collected data (cefazolin-related parameters and concentration–time profiles of cefazolin) in GastroPlus. Some input data that could not be obtained from the literature were predicted using ADMET Predictor v13.0. A schematic flow of the approach followed to develop, verify, and apply the cefazolin PBPK model is illustrated in Figure 1.

An extensive literature review was conducted to identify published clinical studies for the development of the PBPK model of cefazolin in various populations, including healthy adults and pediatric individuals, as well as special populations with varying renal functions. Cefazolin (C_14_H_14_N_8_O_4_S_3_) has a molecular weight of 454.51 g/mol and a log *P* value of −0.24 [17]. Other parameters regarding cefazolin, including plasma protein binding, the blood-to-plasma ratio, and tissue distribution, used in building the initial model are presented in Table 1. The unbound fraction was set to 11% [5].

Cefazolin is predominantly excreted in urine, up to 80% [4]. The elimination half-life (t_1/2_) is 1.8 h in adults and 1.95 h in children, and it is prolonged in cases of renal impairment [5]. The volume of distribution (Vss) is 0.193 ± 0.064 L/kg in adults [18], while in children aged 3 to 12 years it is 0.133 ± 0.015 L/kg [19].

Prior to PBPK model development, non-compartmental analysis was performed using PKPlus^TM^ to characterize the pharmacokinetic profile of cefazolin based on the available clinical concentration–time profiles from the literature and confirm that the PBPK model adequately reproduced the observed pharmacokinetic behavior of cefazolin.

In addition, compartmental pharmacokinetic modeling was also conducted using PKPlus^TM^ to identify the structural model that best described the data. The identified PK profile was then used to ensure consistency between the compartmental analysis and the PBPK model simulations. Following intravenous IV bolus administration, a cefazolin model was first built in a healthy adult population, simulations were performed, and the parameters were integrated and optimized to achieve the best fit with the observed data.

For intravenous administration, cefazolin was introduced directly into the systemic circulation; therefore, gastrointestinal absorption processes were not applicable to the IV simulations. For intramuscular administration, where applicable, drug input was represented through the administered muscle site before entering the systemic circulation. Thus, the route-specific drug input was maintained according to the clinical administration route used in each validation dataset.

In the PBPK model, all tissues are set as perfusion-limited, consistent with cefazolin’s hydrophilic nature and rapid equilibration in well-perfused organs; exceptions include adipose tissue, which is set as permeability-limited due to its low tissue perfusion, limited aqueous space, and slow drug diffusion, and the kidneys, which are also set as permeability-limited, since part of the clearance occurs via transporters that are well represented in permeability-limited models. The Lukacova (Rodgers-single) method is used to determine Kps for perfusion-limited tissues, while the Poulin and Theil extracellular method is used for permeability-limited tissues.

In GastroPlus, tissue distribution was mechanistically represented using individual tissue compartments, with tissue-specific physiological characteristics and drug-related parameters determining the distribution of cefazolin. For perfusion-limited tissues, distribution was primarily governed by tissue blood flow and tissue-to-plasma portioning (K_p_), whereas permeability-limited tissues additionally accounted for the permeability surface area product (P_Stc_) as a determinant of drug transfer between compartments. The unbound fraction and blood-to-plasma ratio were also incorporated as drug-specific inputs influencing distribution and systemic disposition.

Renal elimination of cefazolin was defined in the PBPK model by GFR multiplied by the fraction of unbound drug (GFR × f_up_), along with active tubular secretion modulated by MRP4 and OAT3. MRP4 and OAT3 were incorporated into the model for their role in active renal tubular secretion of cefazolin. K_m_ and V_max_ values for these transporters were retrieved from the literature [20] and were optimized for refinement of the PBPK model as shown in Table 1.

**Table 1:** Inputs used in PBPK model development using GastroPlus™.

Parameter	Value	Reference
Physiochemical properties:		
Molecular weight	454.51	[21]
Log *P*	−0.24	[17]
Log D	3.06 @ pH = 6.5	ADMET predictor
Solubility	350 mg/mL @pH = 7	[22]
Acid pKa (carboxylic acid)	2.1	[22]
Blood/plasma ratio (R_bp_)	0.55	[23]
F_up_ (%)	11	[24]
Transporter kinetics:		
MRP4		
K_m_	36.77 μg/mL	[20,25]
V_max_	0.0225 nmol/min/mg Prot. (0.033 mg/s/mg trans.)	Fitted [26]
OAT3		
K_m_	53.18 μg/mL	[25]
V_max_	0.136 nmol/min/mg Prot. (0.24 mg/s/mg trans.)	Fitted [26]

Accordingly, the major elimination pathway incorporated in the model consisted of renal filtration of unbound cefazolin together with transporter-mediated renal secretion. Changes in renal function and transporter activity were subsequently incorporated to represent the altered renal disposition of cefazolin in the different stages of renal impairment. No additional major metabolic clearance pathway was introduced as a principal elimination mechanism in the model.

The model therefore provided a mechanistic representation of the major ADME processes relevant to cefazolin, including route-specific systemic drug input, physiological tissue distribution, renal filtration, and transporter-mediated renal elimination. However, a formal whole-body mass balance assessment based on experimental recovery of the total administered drug was not performed, and model performance was evaluated by comparison of simulated concentration–time profiles and pharmacokinetic parameters with available clinical observations across the developed populations.

### 2.3. Parameter Sensitivity Analysis

Parameter sensitivity analysis (PSA) was performed to identify the parameters that have the greatest influence on model outputs. It was performed twice: the first to test which properties have the highest effect on model outputs, and the second to check the main PK parameters responsible for cefazolin clearance from the body. PSA was conducted primarily to test model robustness and identify the key factors influencing cefazolin PK. A ten-fold range (0.1-fold to 10-fold relative to the baseline) and a two-fold range (0.5-fold to 2-fold relative to the baseline) were used to test the parameters’ effects on C_max_ and AUC. To guarantee equal representation throughout the whole range of variation, ten test values were assessed for each parameter using logarithmic spacing.

### 2.4. Adult PBPK Model Development

Pharmacokinetic data from two clinical studies in a healthy adult population with normal renal function who received cefazolin via intravenous administration were used to develop the adult PBPK model [26,27]. The mean demographic characteristics from all the studies were used to determine the representative adult population, which included an age of 32 years, a body weight of 79 kg, and a body mass index (BMI) of 25.38 kg/m^2^.

Intravenous infusion and intravenous bolus of 1000 mg cefazolin were used to model cefazolin administration in the chosen studies. GastroPlus has a complete PBPK distribution model. All tissues were set as a perfusion-limited model, except for adipose tissue, which was set as permeability-limited due to the hydrophilicity of the drug, meaning that it will be primarily ionized at physiological pH and thus have limited partition and crossing to tissues. In addition to adipose tissue, kidneys were also set as permeability-limited to obtain better expressions for the transporters. Active tubular secretion and the glomerular filtration rate (GFR) were used to mechanistically model renal elimination. Renal filtration is represented by multiplication of the glomerular filtration rate by the unbound fraction of cefazolin (GFR × F_up_).

Active tubular secretion was incorporated via organic anion transporter 3 (OAT3), responsible for the basolateral uptake of the drug, and multidrug resistance-associated protein 4 (MRP4), responsible for apical efflux in the kidneys. Renal transporter expression levels were retained at their default values from the GastroPlus human physiological database, and transporter-mediated renal clearance was described using Michaelis–Menten kinetics, with V_max_ and K_m_ parameters specified for both renal transporters.

To develop the PBPK model of cefazolin for healthy individuals, renal clearance was mechanistically incorporated by integrating both glomerular filtration rate along with active tubular secretion via renal transporters OAT3 and MRP4. Moreover, our PBPK model integrated a description of the saturable renal secretion of cefazolin via OAT3 and MRP4 transporters. Furthermore, the active OAT3-mediated uptake of cefazolin was assumed to be the rate-limiting step in cefazolin clearance from the body, which prevents accumulation in renal proximal tubules, and this is consistent with the absence of renal toxicity of cefazolin, unlike other antibiotics [7].

Renal tubular secretion of cefazolin was incorporated into the PBPK model through the renal transporters OAT3 and MRP4. The corresponding transporter kinetic parameters, including K_m_ and V_max_, were incorporated into GastroPlus ^®^ based on previously reported values and model fitting to clinical pharmacokinetic data [23,28]. Sensitivity analysis was subsequently performed by systematically varying the K_m_ and V_max_ values of the renal transporters to assess their influence on cefazolin pharmacokinetic parameters, including C_max_ and AUC_0-∞_. The resulting sensitivity profiles were used to characterize the contribution of the individual renal transport processes to cefazolin disposition and to support selection of the transporter parameters used in the final model.

### 2.5. Adult CKD PBPK Model Development

The impact of renal impairment on adult patients was examined using the validated adult model. Active tubular secretion and renal filtration were both modified to express the CKD population. Renal transporter capacity must be further reduced and scaled by a particular factor based on the stage of renal impairment (RI) in order to reflect decreased renal secretory capacity with declining renal function. This factor was mainly applied to OAT3-mediated clearance and was computed using Equation (1).

(1) $$S_{\mathrm{GFR}}=\frac{{\mathrm{GFR}}_{\mathrm{CKD}}}{{\mathrm{GFR}}_{\mathrm{Healthy}}}$$

Since S_GFR_ is the scaling factor, GFR_CKD_ is the GFR for each CKD stage in GastroPlus for the built model, and GFR_Healthy_ is the GFR of the healthy pediatric that was already validated.

For mild and moderate stages, OAT3 capacity was assumed to decline proportionally with GFR [29]; so, transporter expression is multiplied by the scaling factor to represent transporter capacity in patients with mild and moderate RI. Transporter activity in severe and end-stage renal disease (ESRD) populations was further scaled by a factor 0.5 to account for CKD-associated reductions in transporter-mediated renal secretion. Tan et al.’s (2022) methodical analysis of clinical pharmacokinetic data for 33 OAT1/3 substrates throughout various phases of chronic renal disease served as the basis for this scaling [29]. Active tubular secretion is thought to decrease in direct proportion to glomerular filtration rate (GFR) under the intact nephron hypothesis (INH) [30,31]. The study showed that in severe CKD, the decrease in OAT1/3-mediated secretory clearance was more than that expected by GFR alone, with an average of 0.50 ± 0.29 for the ratio of secretory clearance drop to GFR decline [29]. These results led to the recommendation of PBPK simulations of severe CKD and ESRD to reduce transporter function by an additional 50% beyond GFR scaling. In order to account for the documented impairment in tubular secretion, transporter activity in the current model was scaled appropriately. The effects of renal impairment were demonstrated as a reduction in transporter capacity rather than changes in transporter affinity; hence, transporter affinity (K_m_) was kept constant across all CKD stages.

This strategy was based on the mechanistic function of OAT3 as the main rate-limiting step for active renal secretion for cefazolin [32]. MRP4 activity in the CKD populations did not undergo disease-specific scaling. There is currently no indication of a progressive drop in MRP4 function with CKD severity, in contrast to what we observed with OAT3. Despite reports of temporary suppression by several uremic toxins, MRP4 mRNA expression or transport activity does not appreciably change [33,34]. Therefore, transporter activity was kept constant in the current model due to the lack of clinical or quantitative evidence suggesting CKD-dependent impairment of MRP4.

Reductions in the hematocrit observed in patients with moderate and severe renal impairment were also considered when estimating cefazolin pharmacokinetics and plasma concentrations. An adjusted blood/plasma ratio (R_bp_) is calculated via Equation (2) [35]:

(2) $$R_{\mathrm{bp},\mathrm{adj}}=\frac{{\mathrm{Hct}}_{\mathrm{adj}}}{{\mathrm{Hct}}_{\mathrm{healthy}}}\times \left(R_{\mathrm{bp},\mathrm{healthy}}-\left(1-{\mathrm{Hct}}_{\mathrm{healthy}}\right)\right)+(1-{\mathrm{Hct}}_{\mathrm{adj}})$$

where R_bp,adj_ is the adjusted blood/plasma ratio in CKD patients, Hct_adj_ is the hematocrit in CKD patients, Hct_healthy_ is the hematocrit in a healthy population, and R_bp,healthy_ is the blood/plasma ratio in a healthy population.

### 2.6. Pediatric PBPK Model Development

The validated adult PBPK model was scaled down to pediatric populations using PEAR^TM^ physiology in GastroPlus. Age-dependent changes in anatomical and physiological parameters, including blood flow to various organs and organ sizes, were automatically scaled according to the built-in pediatric physiology database to represent healthy pediatric subjects across the relevant age and weight ranges.

Renal clearance via glomerular filtration rate (GFR) was scaled down according to age-dependent physiological scaling and plasma protein binding via Equation (3).

(3) $${\mathrm{Cl}}_{\mathrm{GFR},\mathrm{child}}=\frac{{\mathrm{GFR}}_{\mathrm{Child}}}{{\mathrm{GFR}}_{\mathrm{Adult}}}\times \frac{f_{u,\mathrm{child}}}{f_{u,\mathrm{adult}}}\times {\mathrm{Cl}}_{\mathrm{GFR},\mathrm{Adult}}$$

where f_u,child_ is the fraction of unbound drug in children and f_u,adult_ is the fraction of unbound drug in adults. GFR is the glomerular filtration rate. A similar method was used in different studies [36,37].

In addition to filtration, active tubular secretion was also incorporated via renal transporters (OAT3 and MRP4), which were also scaled to pediatrics by adjusting renal transporter expression to reflect age-dependent changes in kidney physiology. Based on reported renal mRNA expression profiles [38], OAT3 expression was modestly reduced by 5% in children compared to adults to reflect minimal ontogenic differences beyond infancy, whereas MRP4 expression exhibited no consistent age dependency from childhood through adulthood, remaining similar to adult expression [38]. Transporter affinity (K_m_) remained constant across all age groups since developmental effects primarily influence transporter abundance rather than the affinity of the substrate.

The permeability surface area product for tissue cells (Pstc) controls passive drug transport across cellular membranes between the extracellular and intracellular compartments in permeability-limited tissues. The age-dependent physiological scaling algorithms in the PEAR^TM^ physiology module of GastroPlus^®^ were used to automatically scale Pstc values in the pediatric PBPK model from the validated adult model. Pstc was modified in accordance with developmental changes in tissue geometry, such as tissue cellular volume and cellular surface area. The intrinsic passive membrane permeability of cefazolin was assumed to remain constant across age groups; as a result, without the need for manual modification of the drug-specific permeability parameters, passive tissue uptake was automatically adjusted to reflect physiological growth and maturation.

### 2.7. Model Development of Pediatric Patients with CKD PBPK 

The PBPK model in healthy pediatrics without RI was scaled to pediatric patients with different stages of renal impairment. We used the PEAR physiology module in GastroPlus to create a single virtual population that represented the demographic average of the pediatric population in order to initially scale the model to pediatrics with RI. The same CKD-related physiological changes that were detailed for the adult CKD PBPK model (Section 2.4) were followed here. In summary, renal transporter expression and activity (OAT3 and MRP4) were updated using the respective CKD stage-specific scaling factors, while age-specific GFR was adjusted according to CKD severity using the same renal function scaling approach. Additionally, the blood–plasma ratio (R_bp_) variations that resulted from adjusting hematocrit values for each stage of CKD were integrated into the model. These changes made it possible to create a pediatric PBPK model that simulates mild, moderate, severe, and end-stage renal disease (ESRD).

### 2.8. Model Validation

#### 2.8.1. Deterministic Model Validation

By visually comparing the simulated plasma concentration–time profiles with the observed data from the published clinical studies, the predictive performance of the created PBPK models was first evaluated. The model’s capacity to accurately depict the cefazolin distribution and elimination phases was assessed.

All studies and dose levels of simulated pharmacokinetic parameters were compared to observed values. Fold errors and the average fold error for the area under the curve from zero to t (AUC_0-t_), the area under the curve from zero to infinity (AUC_0-∞_), and C_max_ were the main metrics used to assess the predictive ability of the model [39]. A total of 13 studies were used in model validation and are summarized in Supplementary Table S2. The observed pharmacokinetic parameters were calculated from the observed concentration data loaded into GastroPlus ^®^ (version 9.9). The observed concentration–time profiles were directly imported into the software, and parameters including the area under the curve from zero to t (AUC_0-t_), the area under the curve from zero to infinity (AUC_0-∞_), and C_max_ were calculated by GastroPlus using the corresponding observed datasets.

Equation (4) shows that fold error was computed as the ratio of simulated data to observed values. Equation (5) was used to compute the average fold error (AFE).

(4) $$\mathrm{FE}=\frac{\mathrm{Simulated}\;\mathrm{values}}{\mathrm{Observed}\;\mathrm{values}}\;$$

where FE is the fold error change:

(5) $$\mathrm{AFE}={10\;}^{\frac{1}{N}\sum \log\left(\mathrm{FE}\right)}$$

where AFE is the average fold error change, and N is the number of observations.

We simulated plasma concentration profiles from single and multiple doses via various administration routes in order to assess the predictive accuracy of the developed models. AFE was computed to evaluate the potential bias between the datasets. Predictions were deemed acceptable if the FE and AFE values fell within a two-fold error range (0.5–2) [39].

#### 2.8.2. Population Simulation

To assess how well the created PBPK models captured inter-individual variability, population simulations were run using the population simulator module within GastroPlus^®^. The demographic information provided in each validation study was used to create virtual populations. Monte Carlo simulations were performed using a cohort of 100 virtual subjects (*N* = 100) per population simulation. Physiological, formulation, and pharmacokinetic parameters were randomly sampled from their predefined parameter-specific distributions to incorporate interindividual variability. For pediatric simulations, age-dependent and physiological dependent parameters were generated using the PEAR physiology framework, with additional variability incorporated according to the predefined parameter distributions. The 90% probability interval and simulated plasma concentration were displayed, and the observed plasma concentration–time data were superimposed for visual comparison. When the simulated 90% probability interval sufficiently covered the observed concentration data, the model’s performance was deemed acceptable.

#### 2.8.3. Goodness-of-Fit Assessment

Goodness-of-fit analysis was carried out for the healthy adult, adult with CKD, and healthy pediatric models in order to quantitively evaluate the agreement between the simulated and observed plasma concentration data. The predicted plasma values for each population were shown against the corresponding observed concentrations from the separate clinical studies that were used to validate the model. The corresponding population dataset contained all available paired observed and predicted concentration levels from the assessed studies. The degree of linearity of the relationship between the predicted and observed plasma concentrations was measured using the coefficient of determination (R^2^). For a visual evaluation of model performance, each GOF graph contained an identity line (y = x), which represents full agreement between predicted and observed concentrations.

### 2.9. Dose Optimization

The PBPK models of pediatrics patients with varying degrees of RI were used to develop model-based dosing recommendations. AUC rise can be used to predict cefazolin exposure in RI patients. Simulations were used to estimate cefazolin PK profiles in those patients compared to children in the clinical study with normal renal function [5]. The appropriate dosage for children with reduced renal function was determined using Equation (6) [40]

(6) $$\mathrm{Recommended}\;\mathrm{Dose}=\frac{\;{\mathrm{Mean}\;\mathrm{AUC}}_{\mathrm{Healthy}}}{{\mathrm{Mean}\;\mathrm{AUC}}_{\left(\mathrm{RI}\right)}}\times \mathrm{Standard}\;\mathrm{Dose}$$

where Mean AUC_healthy_ is the AUC in the healthy pediatric model, Mean AUC_(RI)_ is the AUC in pediatric patients with varying stages of RI, and standard dose is the dose given to healthy pediatric patients.

#### Pharmacodynamic Evaluation of the Optimized Regimens

The simulated plasma concentration–time profiles were used to assess each optimized dosing regimen’s pharmacodynamic performance after dosage optimization. Since the optimization module does not support T > MIC as an optimization objective, pharmacodynamic activity must be evaluated independently after optimization. As cefazolin is used for both the treatment of bacterial infections and perioperative surgical prophylaxis, the assessment was performed using unbound cefazolin concentrations. The unbound concentration was calculated using the 11% unbound fraction specified in the model. For treatment-oriented evaluation, the percentage of each dosing interval during which the unbound plasma concentration remained above the selected MIC of 4 mg/L (%fT > MIC) was calculated by dividing the duration above the MIC by the corresponding dosing interval [11], as described in Equation (7)

(7) $$\%\mathrm{fT}>\mathrm{MIC}=\frac{t_{f}-t_{i}}{\tau }\times 100\;$$

where t_f_ is the time when free plasma concentration drops below 4 mg/L, t_i_ is the time when free plasma concentration initially exceeds the MIC, and τ is the dosing interval.

For surgical prophylaxis, target attainment was evaluated separately by determining whether the unbound cefazolin concentrations remained above the selected MIC continuously throughput the simulated surgical period, from incision to skin closure. A target of 100%fT > MIC during the surgical period was applied based on the perioperative pharmacodynamic approach reported previously [41].

## 3. Results

### 3.1. Non-Compartmental and Compartmental Pharmacokinetic Analysis

Non-compartmental analysis was initially conducted in order to assess the fundamental systemic disposition characteristics following IV bolus administration of 500–1500 mg cefazolin. A terminal elimination half-life of 1.647–3.102 h, an apparent volume of distribution of 0.1372 L/Kg based on weight-normalized scaling, and an area under the curve from zero to infinity (AUC_0-∞_) of 123.9, 260.1, and 315.6 µg·h/mL following 500, 1000, and 1500 mg IV bolus, respectively, were used. The observed kinetic data were subjected to structural compartmental modeling in order further define the underlying distribution and elimination phases.

The plasma concentration–time profile was found to be most accurately described by a two-compartment model. When compared to one or three-compartment models, the two-compartment model consistently produced the lowest Akaike Information Criterion (AIC) and Schwarz Criterion (SC, also known as the Bayesian Information Criterion, BIC) [42]. The drug shows a clear, quick distribution phase into peripheral tissues before going into the terminal elimination phase according to this statical parsimony.

### 3.2. Parameter Sensitivity Analysis (PSA)

**Figure 2:**
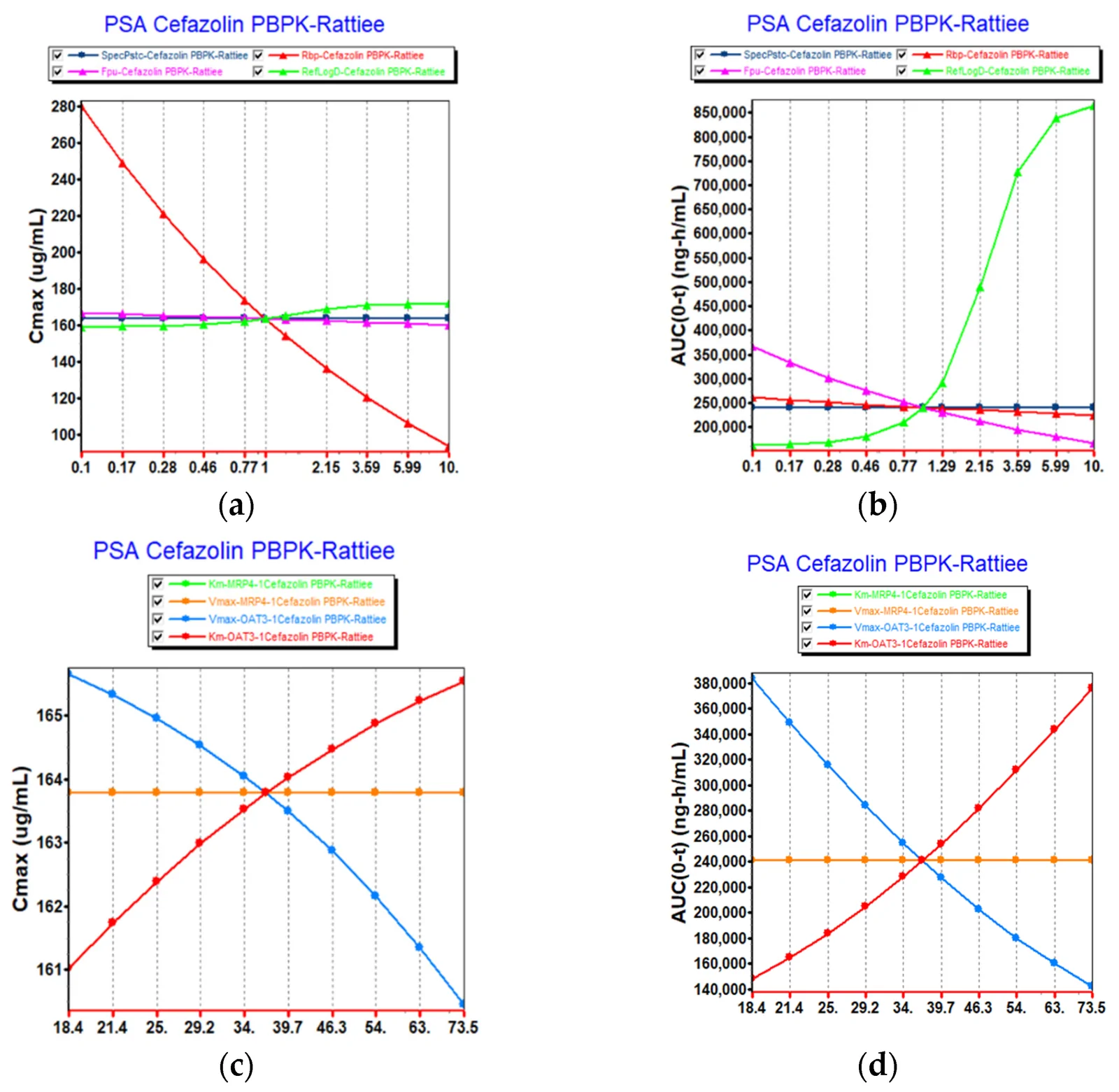
Parameter sensitivity analysis to represent the impact of the unbound fraction in plasma (Fup), the blood-to-plasma ratio (R_BP_), Pstc, and log D on (**a**) C_max_ and (**b**) the AUC, and the impact of transporter kinetics on (**c**) C_max_ and (**d**) the AUC.

Parameter sensitivity analysis identified several key drivers for peak plasma concentrations (C_max_) and total exposure AUC. The reference Log D was most sensitive to total exposure; an increase in the log D value across the range caused a significant, sigmoidal rise in the AUC, but it only slightly increased C_max_. The unbound fraction showed an inverse, moderate association with the AUC, decreasing total exposure as Fup increased but having little effect on peak levels. PSA results for the drug properties can be seen in Figure 2a,b.

The second PSA showed that in contrast to MRP4, OAT3 is the main factor influencing the systemic pharmacokinetics of cefazolin, as can be seen in Figure 2c,d. Systemic exposure and peak plasma concentration varied significantly when K_m_ and V_max_ for OAT3 were changed. In particular, renal active uptake was improved by raising the maximum transport velocity, which resulted in a significant drop in the AUC. On the other hand, changes in MRP4-mediated transporter kinetics produced an entirely flat line for both the AUC and C_max_.

### 3.3. Healthy Adult Model Validation

Eleven different studies were used to validate the healthy adult model. Various routes of administration were tested, including IV bolus, IV infusion, and intramuscular injection. A wide range of doses were incorporated, including 500 mg, 1000 mg, 2000 mg, 3000 mg, and 4000 mg.

**Figure 3:**
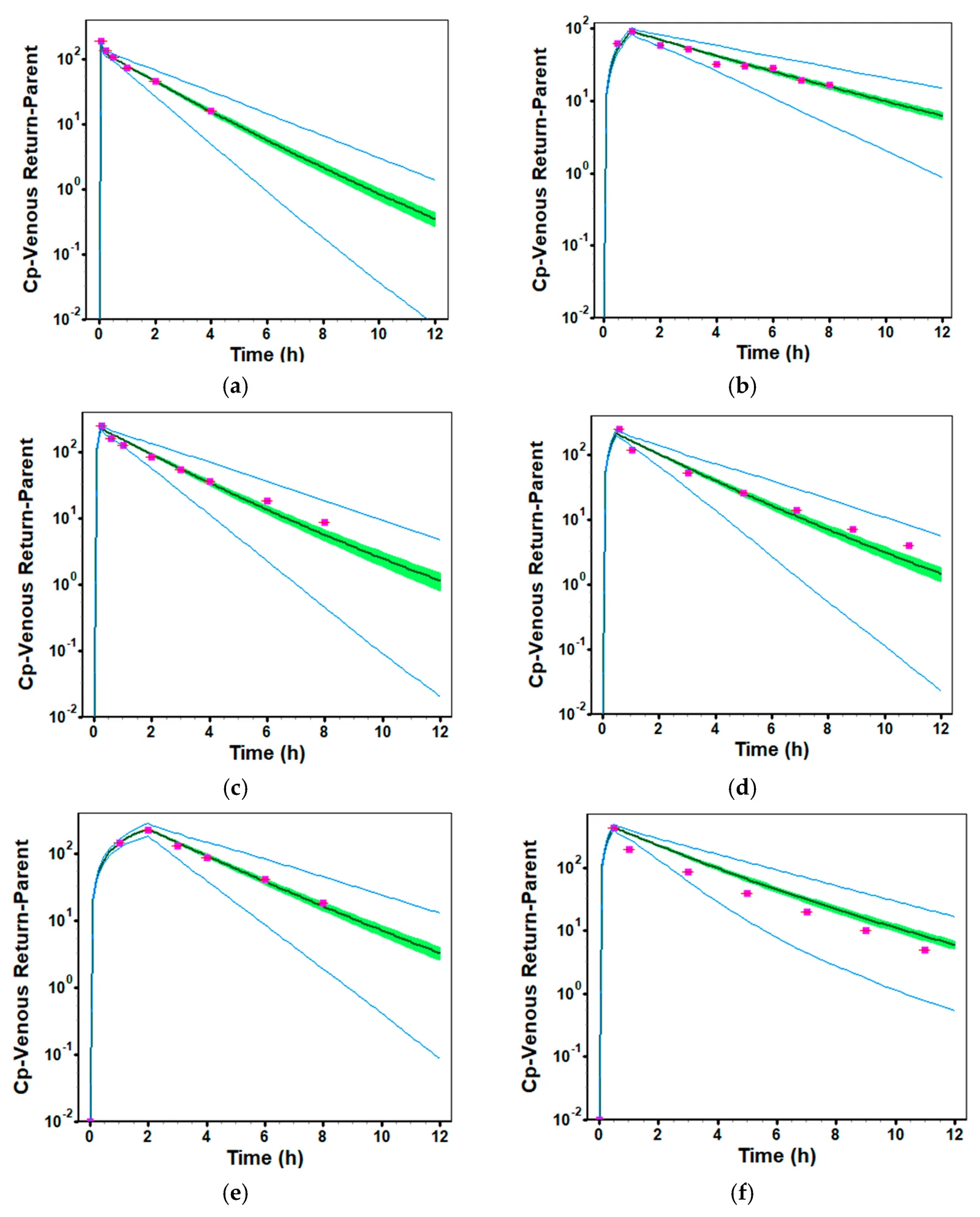
Observed (pink squares) and predicted (black line) plasma concentration−time profiles following cefazolin IV infusion administration in healthy adults: (**a**) 1000 mg [26] (model development), (**b**) 1000 mg [43], (**c**,**d**) 2000 mg [5,44], (**e**) 3000 mg [45] and (**f**) 4000 mg [24]. Blue lines represent 10^th^ and 90^th^ percentiles and green shading represent 90% mean confidence interval.

Strong agreement was shown between the observed clinical values and the simulated plasma concentration–time curves after IV infusion following population simulation to capture the interindividual variations (Figure 3). The clinically observed values were closely mirrored by the expected peak plasma concentration, which fell between 95.527 and 453.53 µg/mL. At the highest evaluated dose of 4000 mg, the model showed slight overprediction of the total systemic exposure with a predicted value of 1138.4 µg·h/mL compared to an observed value of 776.93 µg·h/mL. Nevertheless, the simulation remained well within the widely recognized two-fold error range. For the pharmacokinetic metrics (Table 2), individual fold errors (FEs) ranged from 0.894 to 1.473 and the average fold error (AFE) ranged between 0.999 and 1.152, further supporting the correctness of the prediction model.

**Figure 4:**
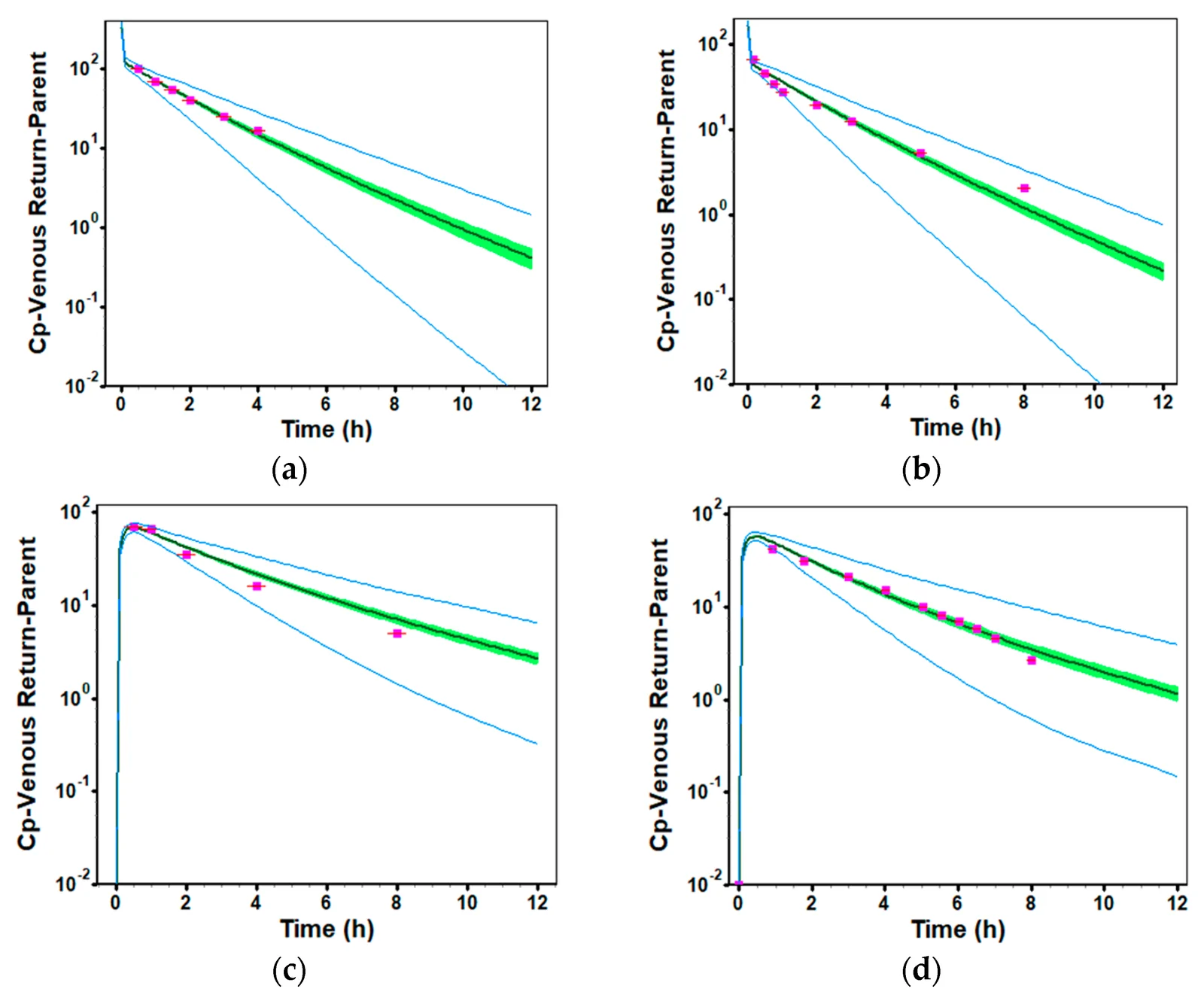
Observed (pink squares) and predicted (black line) plasma concentration−time profiles following cefazolin IV bolus administration in healthy adults: (**a**) 1000 mg (model development) [27], (**b**) 500 mg [47], and (**c**,**d**) 500 mg IM dose [7,50]. Blue lines represent 10^th^ and 90^th^ percentiles and green shading represent 90% mean confidence interval.

For IV bolus, three studies were used for testing, including 500 mg and 1000 mg doses [47,48,49]. FE ranges from 0.1 to 889.795 and AFE ranges from 1.01 to 75.240; their values are shown in Table 3. All values were within the accepted two-fold range. Predicted C_max_ values were 88.654 µg/mL and 162.18 µg/mL following 500 mg and 1000 mg, respectively. They are as expected; C_max_ is expected to double as we double the dose. Similarly, predicted AUC_0-∞_ was 116.58 µg·h/mL and 238.81 µg·h/mL, close to the observed values of 116.83 µg·h/mL and 217.83 µg·h/mL following 500 mg and 1000 mg, respectively. For intramuscular administration, two studies were used to validate our healthy adult model, with FE ranging from 0.987 to 1.246, and all simulations were within the two-fold error range and can be seen in Table 3. AUC_0-∞_ values following 1000 mg IV infusion are consistent with the previous values for the IV bolus with the same dose. Simulated plasma concentration–time profiles for IV bolus and intramuscular administration following the population simulation are shown in Figure 4.

**Figure 5:**
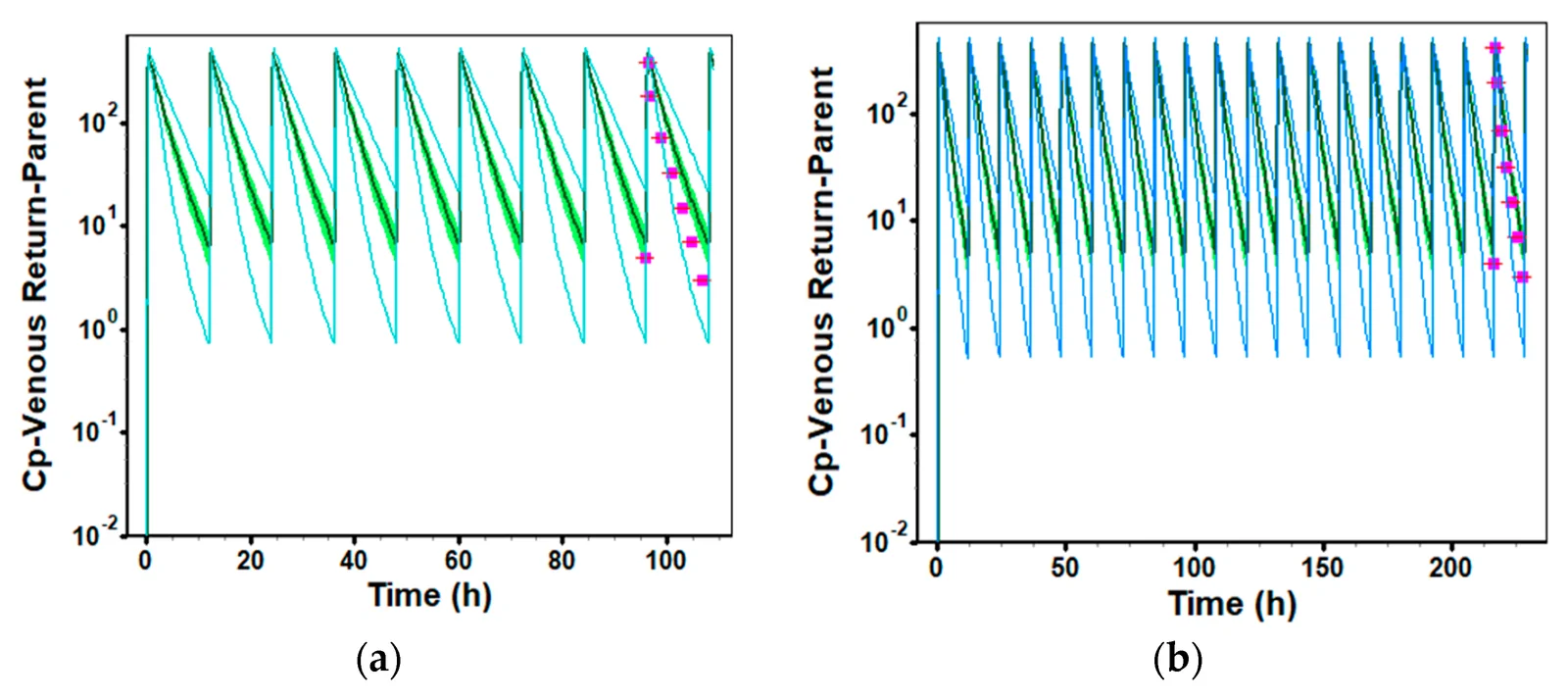
Observed (pink squares) and predicted (black line) plasma concentration−time profiles following multiple-dose cefazolin IV bolus administration in healthy adults: (**a**) 9^th^ dose and (**b**) 19^th^ dose [24]. Blue lines represent 10^th^ and 90^th^ percentiles and green shading represent 90% mean confidence interval.

Other relevant studies involving intravenous administration of multiple cefazolin dose regimens, up to 19 doses, were used to validate the initial PBPK model to ensure that the model could predict not only single-dose but also multiple-dose administration [24]. FE ranges from 1.004 to 1.251 and AFE from 1.117 to 1.154. Their values are shown in Table 4. C_max_ and AUC_0–∞_ following 1 dose, 9 doses, and 19 doses were similar, which shows that there is no significant accumulation of cefazolin in the body following multiple doses in healthy individuals. Simulated plasma concentration–time profiles for multiple IV infusions are shown in Figure 5.

### 3.4. Adult with CKD Model Validation

**Figure 6:**
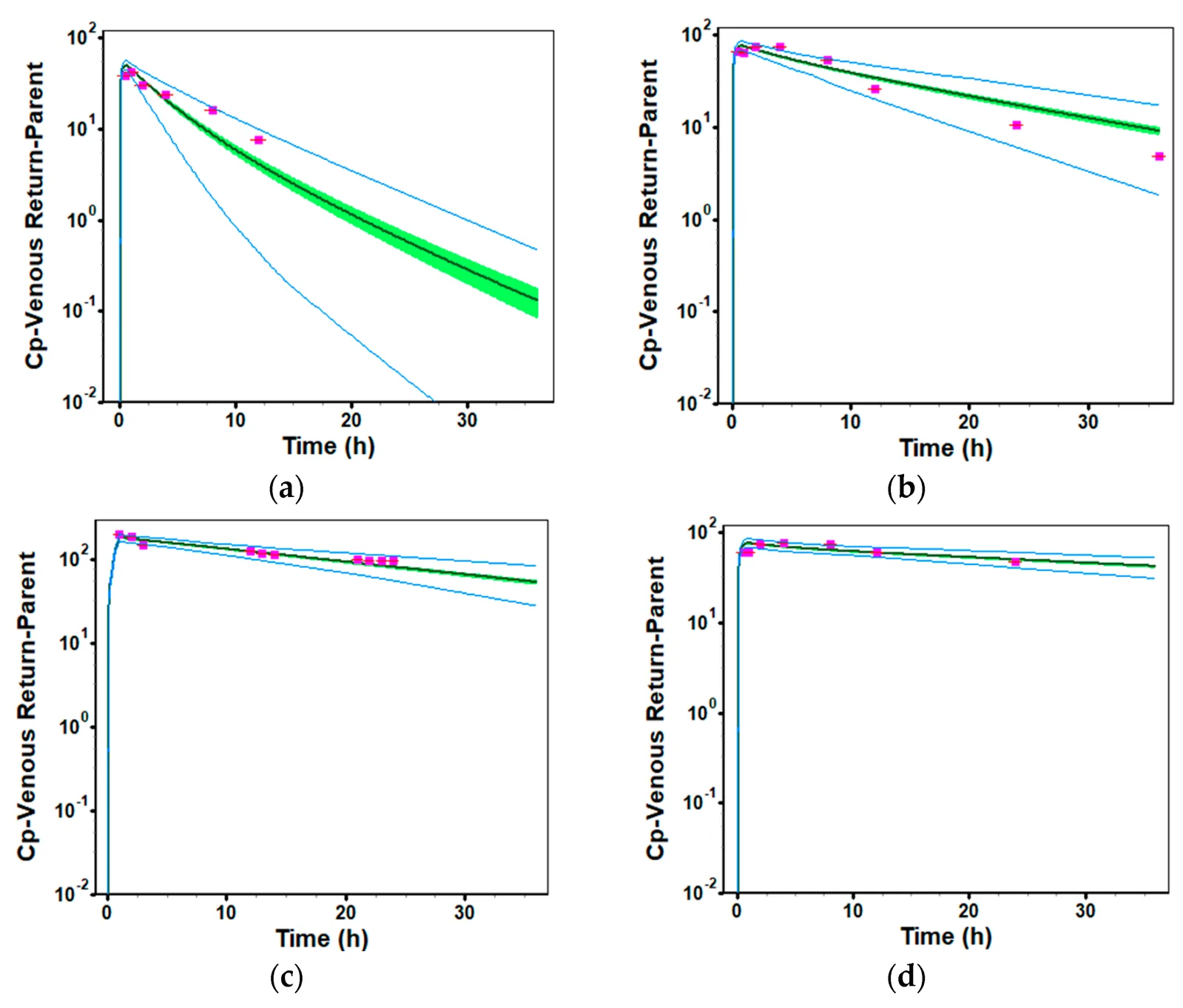
Observed (pink squares) and predicted (black line) plasma concentration−time profiles in adults with varying degrees of renal impairment (**a**) Moderate: 500 mg IM [7]. (**b**) Severe: 500 mg IM [7]. (**c**) Severe: 2000 mg IV infusion [43]. (**d**) End-stage: 500 mg intramuscular [7]. Blue lines represent 10^th^ and 90^th^ percentiles and green shading represent 90% mean confidence interval.

Three studies were used to test the effects of renal impairment in the adult model [7,51,52]. The studies include 2000 mg IV infusion for a stage III (severe CKD) patient, and a 500 mg IM dose for patients with severe and end-stage renal disease. C_max_ was not affected by renal impairment, while AUC_0–∞_ was highly affected by the changes in renal function, which shows higher exposure of the drug in the body for patients with worsening renal function. FE and AFE are shown in Table 5. Moving to more severe stages, we can see that the predicted AUC_0–∞_ in the moderate stage was 223.55 µg·h/mL following a 500 mg cefazolin dose. The predicted AUC_0–∞_ was 1234.5 and 2123.1 µg·h/mL following a 500 mg IM dose in severe and end-stage renal disease, respectively. Simulated plasma concentration–time profiles are shown in Figure 6.

### 3.5. Healthy Pediatric Model Validation

Two separate clinical studies with pediatric patients of various ages and dosage schedules were used to assess the predictive efficacy of the scaled healthy pediatric PBPK model. In the first study, cefazolin was administered as surgical prophylaxis to hospitalized children between the ages of 10 and 12 years who were scheduled for surgery [5]. Two doses were tested in this clinical study based on the patient’s weight as either 1 g or 2 g through 30 min IV infusion regimens. In the second study, children between the ages of 3 and 12 years received a weight-based dose of 25 mg/kg of cefazolin. Inclusion of those two studies enabled us to evaluate the model across a broader pediatric age range and under different dosing conditions.

**Figure 7:**
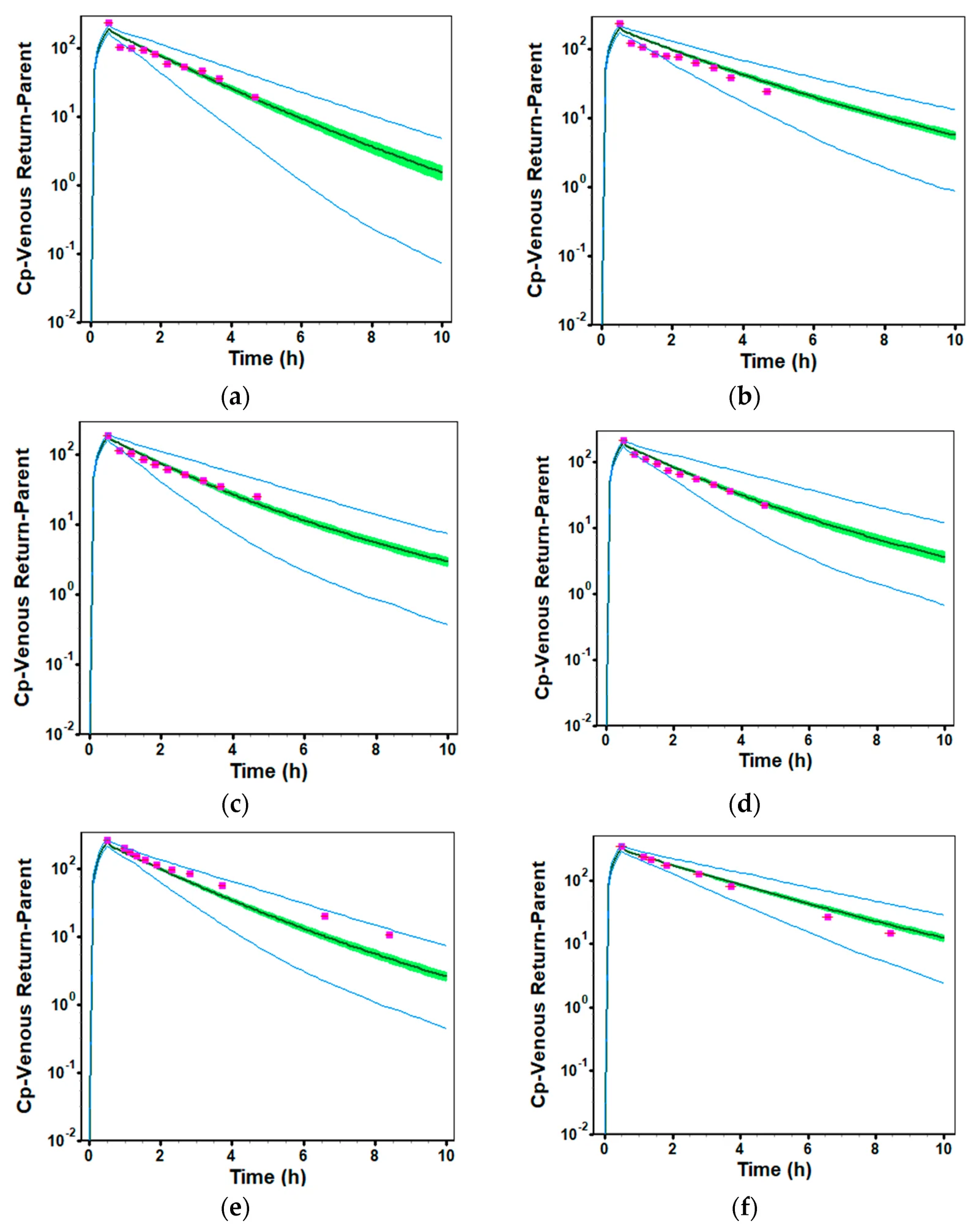
Observed (pink squares) and predicted (black line) plasma concentration−time profiles following cefazolin IV infusion administration in healthy pediatrics. A dose of 25 mg/kg is given as IV infusion [19] for pediatrics aged (**a**) 3 years, (**b**) 4 years, (**c**) 6 years, and (**d**) 9 years; (**e**) 1000 mg as IV infusion for pediatrics aged 10–12 years [5]; (**f**) 2000 mg as IV infusion for pediatrics aged 10–12 years [5]. Blue lines represent 10^th^ and 90^th^ percentiles and green shading represent 90% mean confidence interval.

The model correctly predicted the cefazolin exposure after both 1 g and 2 g dosages for the first clinical study. Following the 1 g and 2 g dosages, the predicted C_max_ values were 251.96 and 327.96 µg/ml, respectively. The corresponding AUC_0-∞_ values were 514.01 and 928.35 µg·h/mL. The predicted and observed pharmacokinetic parameters showed good agreement, with FE in the range of 0.791–1.235 and AFE of 0.907–1.038. For both dosing regimens, the predicted plasma concentration–time profiles closely matched the actual profiles, as shown in Figure 7. Table 6 displays the relevant predicted and observed values along with FE and AFE in the healthy pediatric model.

The predicted pharmacokinetic parameters were assessed using the same fold error range and can be seen in Table 6. The simulated cefazolin concentration–time profile following this dosing schedule is shown in Figure 7. The scaled pediatric model’s capacity to predict cefazolin exposure in wider pediatric population ranges is assessed using those findings.

Overall, the healthy pediatric PBPK model was able to accurately predict cefazolin pharmacokinetics across several pediatric age groups and dose regimens, according to validation against independent clinical studies. The validated pediatric model was further used to build the model of pediatric patients with RI.

### 3.6. Goodness-of-Fit Evaluation

**Figure 8:**
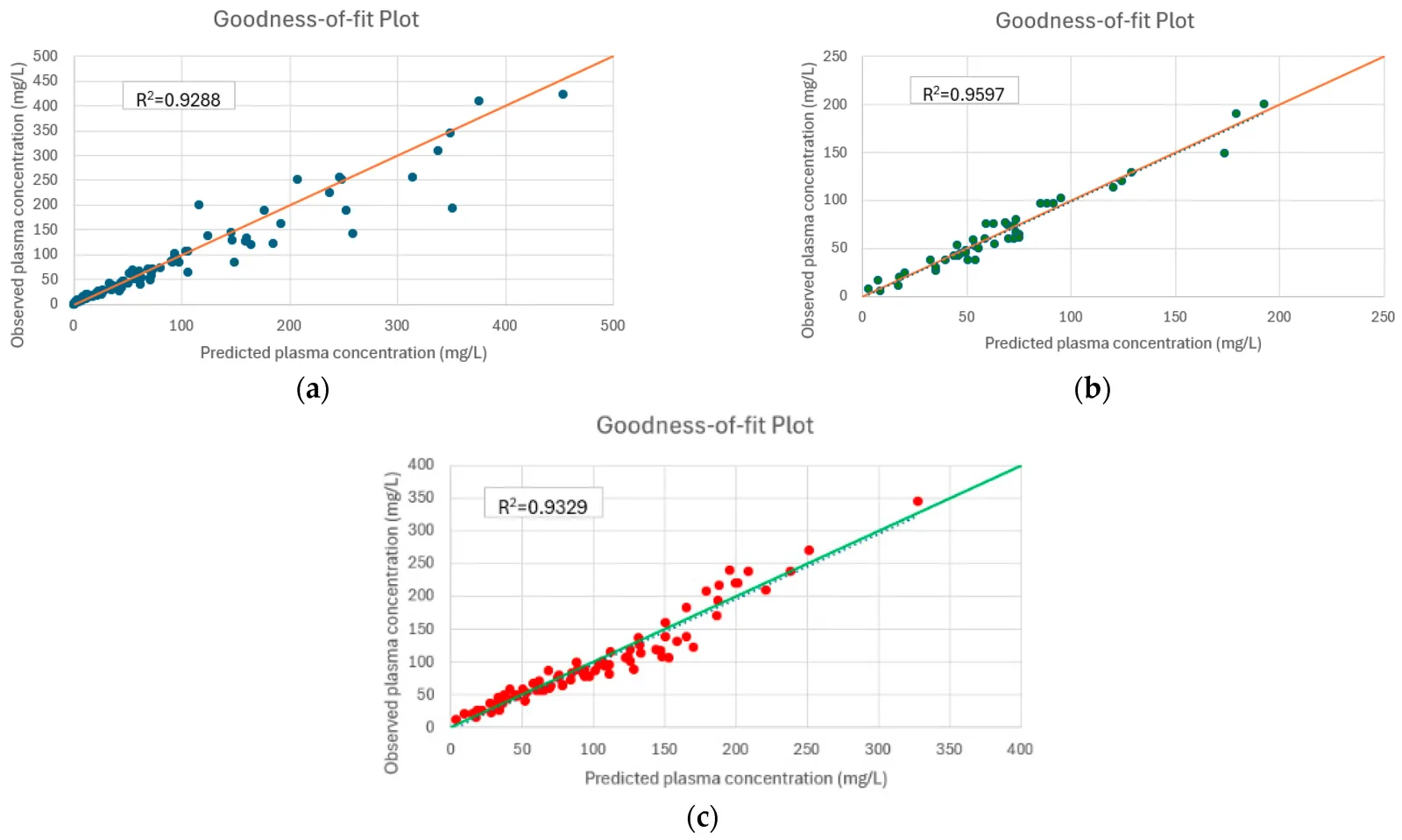
Goodness-of-fit plot, Dots represent the individual observed vs predicted data points, solid line is the line of identity for (**a**) healthy adult model, (**b**) CKD adult model, and (**c**) healthy pediatric model, with their respective coefficient of determination (R^2^).

All developed models showed a significant linear relationship between the predicted and observed plasma concentrations, according to the quantitative goodness-of-fit evaluation (Figure 8). With an R^2^ of 0.9288, the predicted concentrations for the healthy adult model demonstrated a significant correlation with the observed data. The adult CKD model showed the best correlation between the observed and predicted values, with an R^2^ of 0.9597. With an R^2^ of 0.9329, the healthy pediatric model also demonstrated a significant correlation between predicted and observed values. The overall predictive performance of the healthy adult, CKD adult, and healthy pediatric PBPK models is supported by the R^2^ values exceeding 0.92 for all three populations, showing a strong linear association between the simulated and observed plasma concentrations, with the data points typically distributed close to the line of identity.

### 3.7. Pediatric with CKD Model and Dose Optimization

In order to predict the effects of renal impairment on cefazolin exposure and suggest proper dosages in pediatric populations with impaired renal function, in the RI model, the AUC was used as a predictor of cefazolin exposure. This parameter was used for dosage adjustment, as mentioned in Equation (7).

**Figure 9:**
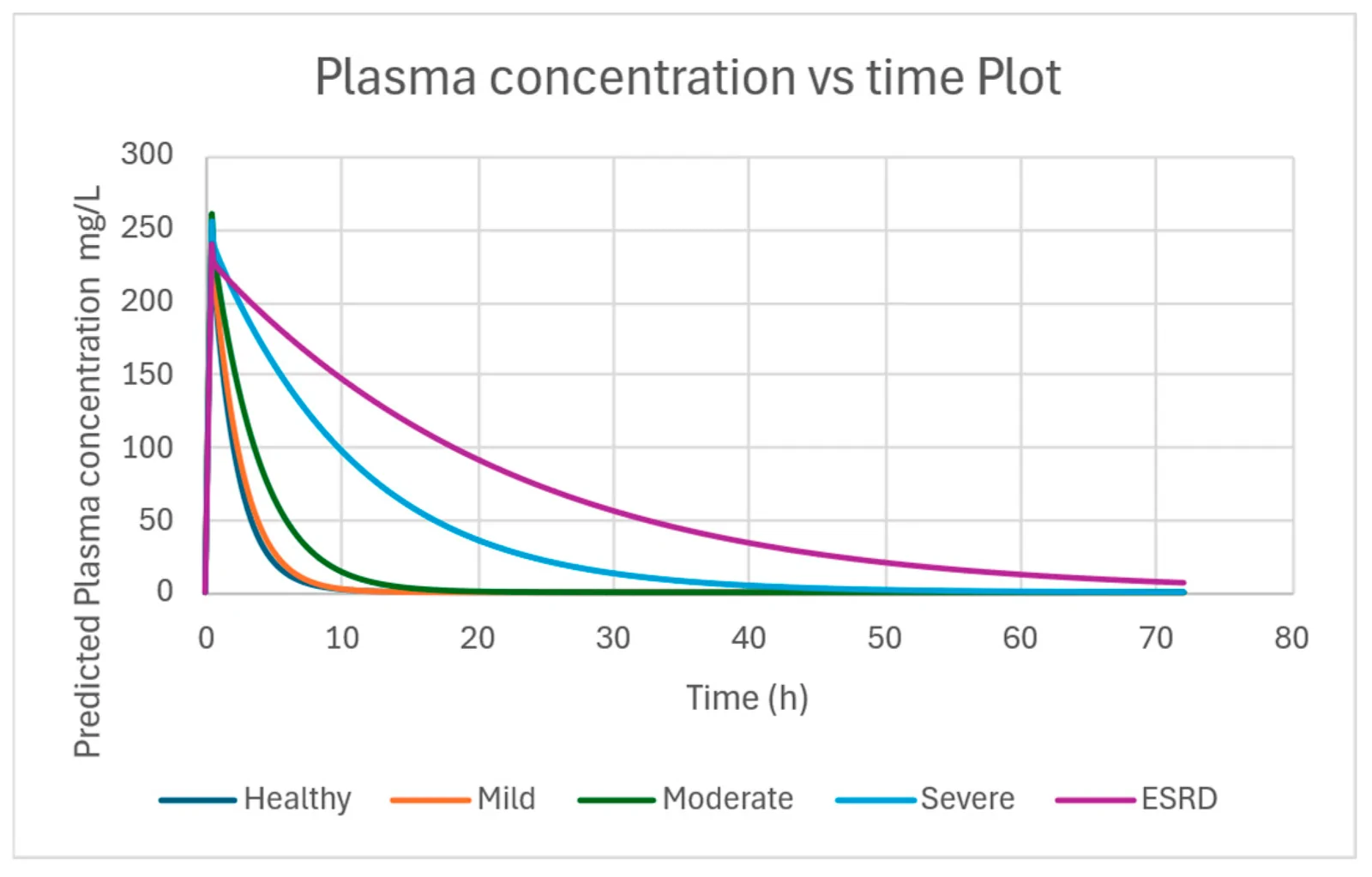
Predicted plasma concentration–time profile following same dose of 1000 mg IV infusion in pediatric patients with varying degrees of renal impairment.

Model-informed dose reductions to 43–90% of the usual pediatric dose were based on the elevation fold of the AUC, as seen in Table 7. For children with moderate and severe RI, the suggested CFT dosages were 15.3 mg/kg BW and 5.41 mg/kg BW. There was no significant increase in C_max_ between the stages of renal impairment. Figure 9 shows the simulations following 1000 mg IV infusion for pediatrics with varying degrees of renal impairment.

Following the optimized doses, the predicted AUC_0-∞_ was 502.28, 485.36, and 493.45 µg·h/mL in moderate, severe and ESRD patients, respectively. Pharmacodynamic performance was subsequently evaluated using unbound cefazolin concentrations. As renal function progressively declined, the duration of unbound cefazolin concentrations remaining above the selected MIC of 4 mg/L increased [54], consistent with the reduced renal elimination of cefazolin. The duration above the MIC was approximately 4.44 h in mild renal impairment, 6.93 h in moderate renal impairment, 19.95 h in severe renal impairment, and 38.76 h in ESRD.

For treatment-oriented assessment, these durations corresponded to approximately 55.5%, 57.8%, 83.1%, and 80.7% fT > MIC for the mild (q8h), moderate (q12h), severe (q24h), and ESRD (q48h) regimens [55], respectively. For surgical prophylaxis, unbound cefazolin concentrations remained above 4 mg/L for approximately 4.5, 7, 20, and 38.8 h in mild RI, moderate RI, severe RI, and ESRD, respectively. Therefore, assuming appropriate preoperative administration, the simulated regimens maintained unbound cefazolin concentrations above the selected MIC throughout a 4 h surgical period in all renal impairment groups [41]. These findings indicate prolonged pharmacodynamic exposure with progressive renal impairment and support the ability of the optimized dose and dosing interval combinations to maintain cefazolin exposure during the simulated perioperative period.

## 4. Discussion

PBPK models representing the pharmacokinetic processes of cefazolin in different populations, including healthy adults with normal kidney function, adults with CKD, and healthy pediatric patients with normal kidney function, were developed and validated. The developed model provided accurate predictions of systemic cefazolin concentrations, as well as the effects of varying degrees of renal impairment on drug exposure and disposition in adults and pediatric patients with CKD. These models showed acceptable inter-patient variability, as all results fell within the acceptable ranges of drug plasma concentrations.

Overall, the predicted and observed data showed a strong correlation, with only very small underprediction in the last phase. Ratios of predicted-to-observed AFE for AUC_0-∞_ and C_max_ were within the two-fold range, indicating the validity of the PBPK model in predicting cefazolin systemic exposure in healthy adults. Fifteen studies were used for model development and validation across different populations including healthy adults, CKD adults and healthy pediatrics (Supplementary Table S2). The prediction of exposures was consistent and confirmed across a wide range of doses including 500 mg, 1000 mg, 2000 mg, 3000 mg and 4000 mg via different routes of administration such as IV infusion, IV bolus and intramuscular injection. The use of multiple studies with various methods of administration, sampling times and assay techniques could explain the variability between the virtual population and the clinical data; however, they still show a strong correlation with each other [56].

The developed models were validated against different studies; model prediction was found to accurately reflect the observed data given in the literature quite well when the simulated plasma concentration–time profiles were compared to the observed data (Figure 3, Figure 4, Figure 5, Figure 6 and Figure 7). The similarity between the predicted and the observed values of drug concentration–time profiles is indicated by the values of the main parameters AUC_0−t_, AUC0-∞, and C_max_ and can be seen in Table 2, Table 3, Table 4, Table 5 and Table 6. The area under the curve and C_max_ were accurately predicted in all studies, while for C_max_, it was slightly overpredicted in some cases. Calculating C_max_ not long after IV injection may be the primary cause of the difference between the observed and the predicted value for C_max_. The initial sample is usually taken at 10–15 min after administration in most of the clinical studies; however, a high C_max_ is not typically observed, making the predicted values much higher than the observed values.

The incorporation of OAT3-medaited renal transport into the cefazolin PBPK model was supported by previous experimental and clinical evidence. OAT3 (SLC22A8) has demonstrated substantial transport activity toward cephalosporin antibiotics, and cefazolin has been identified as an hOAT3 substrate [57,58]. Clinical evidence also supports a contribution of OAT-mediated tubular secretion to cefazolin elimination, as inhibition of renal organic anion transport by probenecid has been reported to increase cefazolin systemic exposure and prolong its serum half-life [7,59]. These findings provide mechanistic support for incorporating OAT3-mediated renal transport into the model. The sensitivity analysis performed in the present study further evaluated the influence of OAT3 and MRP4 kinetic parameters on cefazolin exposure.

Once the model was developed and validated in healthy subjects, it was extrapolated to adults with CKD. Using PEAR^TM^ physiology, we changed the health status to different stages of CKD, after which automated changes were made to the model to match the anatomical and physiological changes observed in CKD patients. In addition to these changes, studies show that OAT3 capacity is affected by the reduction in GFR. For patients with mild and moderate CKD, a proportional reduction in OAT3 expression and capacity was observed based on the reduction in GFR, while in severe and end-stage renal disease, in addition to the proportional reduction, it was suggested to multiply the values by a factor of 0.5 to represent the effects on RI in these two stages [29]. Therefore, we manually adjusted the capacity for OAT3 based on the GFR for each group. The decline in GFR along with transporter expression was not sufficient to describe cefazolin pharmacokinetics in CKD patients, especially those in moderate and severe stages. In such patients, a reduction in the hematocrit is observed, so the blood/plasma ratio needs to be adjusted to achieve better results [35]. Nevertheless, by applying these modifications, the predicted pharmacokinetic profiles for the patients showed high agreement between the predicted and observed clinical profiles, with slight overprediction in the terminal phase. The models showed FE and AFE within the two-fold error range.

Compartmental analysis of the predicted drug concentration–time profiles showed that most of the PK parameters were comparable to the reported values. Elimination half-life was slightly overpredicted in some cases; however, it was within the acceptable range. The developed adult model, after it was validated, was scaled for adults suffering from varying degrees of renal impairment according to KDIGO 2025 classifications [60]. Data from three clinical studies were used to verify the development of the CKD model in adult patients. The model was satisfactorily validated by predicting cefazolin exposure in patients with RI (Table 5). We observed that the severity of renal impairment increases exposure to cefazolin, in AUC_0-∞_ values ranging from 169.99 µg·h/mL in healthy patients to 1234.5 µg·h/mL in patients with severe RI following a 500 mg IM dose. Patients with mild renal impairment have a similar profile to the healthy adult model, so no dose adjustment is required, and dose adjustments are recommended when renal function is less than 55 mL/min based on the drug label [12]. The high increase in cefazolin exposure in moderate and severe stages demonstrates the need for dose reduction to normalize drug exposure and avoid the risk of cefazolin accumulation. The developed CKD model was used for dose optimization after it was validated.

It should be noted that there are variations in GFR values within the same stage of renal impairment [60], which will affect transporter expression, along with variations in age between patients in each stage. Using the Population Simulator, one of the GastroPlus tools, addresses this issue by capturing the inter-individual variability within our virtual populations. It shows that our population falls within the 10th and 90th percentiles, as shown in Figure 2, Figure 3, Figure 4, Figure 5 and Figure 6.

The verified healthy adult model was also scaled to pediatrics, and PEAR^TM^ physiology was used to apply different age-related physiological changes. For the primary parameters AUC_0−t_, AUC_0-∞_, and C_max_, the ratio of observed to projected values was within the acceptable range (Table 6), and its validity was demonstrated by accurately predicting cefazolin exposure in children (Figure 7).

The strong agreement between predicted and observed cefazolin plasma concentrations was shown by the goodness-of-fit analysis for all assessed populations. R^2^ values of 0.9288, 0.9597, and 0.9329 for the healthy adult, adult CKD, and healthy pediatric models, respectively, demonstrated that the models sufficiently represented the reported concentration range across both healthy and renally impaired populations, as seen in Figure 8. The CKD models showed the strongest association, indicating that the cefazolin PBPK framework should include modifications relating to renal impairment.

The developed model’s predictive accuracy is further supported by the close distribution of data around the identity line and the significant correlations found across the three populations. However, as correlation by itself cannot evaluate systematic prediction bias, R^2^ was interpreted in conjunction with fold error and AFE data. All of these results point to the developed PBPK model’s suitability for characterizing cefazolin disposition in CKD patients and healthy adults and pediatrics.

The validated pediatric model was scaled to test the combined effects of age and renal impairment on the concentration–time profiles of cefazolin. Since C_max_ occurs in the early phases following dose administration, it will not be impacted by renal function as serum concentration changes in later phases; so, it did not change significantly as the severity of renal impairment changed. As seen in Table 7, AUC_0-∞_ increased as the severity increased, indicating that the drug will accumulate in the body and that the patient will be exposed to higher concentrations due to worsening kidney function even if administering a similar dose. Based on those values, an adjusted dose is required.

Knowing that cefazolin is a β-lactam antibiotic exhibiting time-dependent antibacterial activity [61], this property was incorporated into the optimization strategy. Optimized dosing regimens were selected to maintain plasma concentrations above the minimum inhibitory concentration (MIC) for a sufficient proportion of the dosing interval, which is 4 µg/mL for most common infections, though it can be higher in severe infections [54]. Pharmacodynamic efficacy is mainly correlated with the time interval during which the free concentration remains above the MIC (%fT > MIC), which should exceed 40–50% of the total dosing interval depending on the organism, infection, and clinical context [62].

Therefore, to ensure the optimal %fT > MIC target, dose adjustments were evaluated in special populations to ensure safe and effective dosing strategies. Dose adjustments were made by modifying the initial dose while maintaining clinically feasible administration schedules and maintaining its concentration above MIC. These regimens were evaluated by comparing the optimized profiles with those obtained in pediatric subjects with normal renal function, ensuring an adequate amount with minimal accumulation in patients with renal impairment. Pharmacodynamic target attainment was successfully maintained while avoiding unnecessary exposure due to the implemented dosing strategies, which included interval extension alone for mild RI and a combination of dosage reduction and interval extension for moderate-to-severe RI patients [55].

When evaluated using the unbound cefazolin concentrations, the resulting %fT > MIC values were 55.5%, 57.8%, 83.1% and 80.7% for mild RI, moderate RI, severe RI, and ESRD, respectively. The increasing duration above the MIC with worsening renal function reflects the progressive reduction in cefazolin elimination and demonstrates the influence of renal function on the duration of pharmacodynamic exposure. The interpretation of these findings should distinguish treatment from surgical prophylaxis. For treatment, the calculated %fT > MIC values describe the proportion of each dosing interval during which unbound cefazolin concentrations exceeded the selected MIC. Although the mild RI regimen resulted in a lower %fT > MIC than more severe RI groups, the prolonged exposure observed with worsening renal impairment demonstrates the expected pharmacokinetic sequence of reduced renal clearance. For surgical prophylaxis, the relevant consideration is the maintenance of adequate unbound cefazolin concentrations throughout the surgical period rather than throughout the entire dosing interval. In the present simulations, unbound cefazolin concentrations remained above 4 µg/mL for approximately 4.5 h, 7 h, 20 h, and 38.8 h in mild RI, moderate RI, severe RI, and ESRD, respectively. Thus, assuming appropriate timing of administration, the optimized regimens maintained concentrations above the selected MIC throughout the simulated 4 h surgical period in all RI groups. This supports their potential suitability for maintaining perioperative antimicrobial exposure while accounting for the prolonged elimination associated with renal impairment [41].

Importantly, the dose reductions and interval extensions were intended not only to maintain pharmacodynamic exposure but also to limit unnecessary cefazolin accumulation in patients with impaired renal function. Excessive cefazolin exposure has been associated with neurological adverse effects, including seizures, particularly in patients with substantial renal dysfunction [6,63]. Overall, these findings demonstrate the utility of the PBPK approach for integrating changes in renal function into cefazolin dosing and for exploring dose and interval regimens across different degrees of pediatric renal impairment.

There are some limitations in the current study. A major limitation is the limited availability of clinical data on cefazolin pharmacokinetics in pediatric patients with varying degrees of renal impairment, which restricted independent validation of the final pediatric RI model and clinical confirmation of the optimized dosing regimens. In addition, the model was developed and evaluated using pediatric data from children aged 3–12 years; therefore, the findings should not be generalized to younger children, particularly infants, without further evaluation. Nevertheless, the predicted pharmacokinetic parameters of the virtual pediatric populations were consistent with published pediatric data. Further clinical studies are warranted to evaluate cefazolin pharmacokinetics and confirm the proposed dose and dosing interval adjustments in children with different degrees of renal impairment, particularly in younger age groups.

## 5. Conclusions

A cefazolin PBPK model was developed and validated to characterize cefazolin exposure in pediatric populations with varying degrees of renal impairment. The model was subsequently applied to optimize cefazolin dose and dosing intervals, with the aim of maintaining appropriate exposure while minimizing excessive accumulation associated with reduced renal clearance. Interval extension alone was sufficient for mild RI, whereas increasing renal impairment required both dose reduction and interval extension. The optimized regimens corresponded to an approximately 43% reduction in the standard dose for 12 h in moderate RI, an 80% reduction in the standard dose administered for 24 h in severe RI, and an approximately 90% reduction administered for 48 h in ESRD.

Pharmacodynamic evaluation was performed using unbound cefazolin concentrations following dose optimization. For treatment-oriented assessment, the resulting %fT > MIC values were 55.5%, 57.8%, 83%, and 80.7% for mild RI, moderate RI, severe RI, and ESRD, respectively, using the selected MIC of 4 µg/mL. For surgical prophylaxis, the optimized regimens maintained unbound concentrations above the MIC for 4.5, 7, 20, and 38.8 h in mild RI, moderate RI, severe RI, and ESRD, respectively, throughout the simulated 4 h surgical period. These findings demonstrate the ability of PBPK modeling to integrate renal function-related changes in cefazolin disposition and to support individualized dose and interval selection in pediatric patients with renal impairment. However, these optimized regimens represent model-based extrapolations requiring clinical confirmation, and their applicability is limited to the pediatric age range of 3–12 years represented in this study. Further clinical investigation is warranted, particularly in younger children and pediatric patients with renal impairment.

## Figures and Tables

**Table 2:** FE and AFE after IV infusion in healthy adults.

Dose and Route	AUC_0-t_ (µg·h/mL)		AUC_0-∞_ (µg·h/mL)		C_max_ (µg/mL)		Reference
	Observed	Predicted	Observed	Predicted	Observed	Predicted	
1000 mg, IV infusion 5 min	231.47	243.6	261.57	243.82	188.9	176.47	[26]
		FE = 1.052		FE = 0.932		FE = 0.934	
1000 mg, IV infusion 1 h	328.09	359.71	436.7	423.64	92.608	95.527	[43]
		FE = 1.096		FE = 0.970		FE = 1.032	
2000 mg, IV infusion 15 min	462.77	489.19	486.39	497.92	250.02	248.1	[5]
		FE = 1.057		FE = 1.024		FE = 0.992	
2000 mg, IV infusion 30 min	476.86	495.02	491.22	496.19	250.81	224.11	[44]
		FE = 1.038		FE = 1.010		FE = 0.894	
2000 mg, IV infusion 30 min	489.25	561.21	503.55	568.22	256	246.28	[24]
		FE = 1.147		FE = 1.128		FE = 0.962	
3000 mg, IV infusion 30 min	570.75	812.2	582.29	822.84	309	338.25	[24]
		FE = 1.423		FE = 1.413		FE = 1.095	
3000 mg, IV infusion 2 h	729.5	752.08	774.63	757.25	225	238.26	[45]
		FE = 1.031		FE = 0.978		FE = 1.059	
4000 mg, IV infusion 30 min	762.5	1123.4	776.93	1138.4	423	453.53	[24]
		FE = 1.473		FE = 1.465		FE = 1.072	
4000 mg, IV infusion 1 h	916.16	1046.6	935.94	1061.6	409.83	398.98	[46]
		FE = 1.142		FE = 1.134		FE = 0.974	
AFE		1.152		1.103		0.999	

**Table 3:** FE and AFE after IV bolus and intramuscular administration in healthy adults.

Dose and Route	AUC_0-t_ (µg·h/mL)		AUC_0-∞_ (µg·h/mL)		C_max_ (µg/mL)		Reference
	Observed	Predicted	Observed	Predicted	Observed	Predicted	
1000 mg, IV bolus	211.78	208.42	217.83	238.81	102.6	162.18	[27]
		FE = 0.984		FE = 1.096		FE = 1.581	
500 mg, IV bolus	110.19	114.79	116.83	116.58	67.31	88.654	[47]
		FE = 1.042		FE = 0.998		FE = 1.317	
1000 mg, IV bolus	202.16	208.61	244.37	238.86	98.802	177.38	[48]
		FE = 1.032		FE = 0.978		FE = 1.795	
1000 mg, IV bolus	223.37	235.04	228.9	238.81	199.5	177.38	[49]
		FE = 1.057		FE = 1.043		FE = 0.889	
500 mg, Intramuscular	194.55	223.88	211.56	244.75	70	69.122	[7]
		FE = 1.151		FE = 1.157		FE = 0.987	
500 mg, Intramuscular	131.5	160.53	136.45	169.99	42.137	48.509	[50]
		FE = 1.221		FE = 1.246		FE = 1.151	
AFE		1.078		1.075		1.240	

**Table 4:** FE and AFE after multiple IV infusion doses in healthy adults.

Dose and Route	AUC_0-t_ (µg·h/mL)		AUC_0-∞_ (µg·h/mL)		C_max_ (µg/mL)		Reference
	Observed	Predicted	Observed	Predicted	Observed	Predicted	
4000 mg, IV infusion 30 min (9)	913.75	1143.3	920.83	1143.28	381	457.12	[24]
		FE = 1.251		FE = 1.242		FE = 1.199	
4000 mg, IV infusion 30 min (19)	1155.3	1143.3	1138.3	1143.2	412	457.12	[24]
		FE = 1.011		FE = 1.004		FE = 1.109	
AFE		1.125		1.117		1.154	

**Table 5:** FE and AFE after cefazolin doses in adults with varying degrees of renal impairment.

Dose and Route	CKD Stage	AUC_0-t_ (µg·h/mL)		AUC_0-∞_ (µg·h/mL)		C_max_ (µg/mL)		Reference
		Observed	Predicted	Observed	Predicted	Observed	Predicted	
500 mg, Intramuscular	Moderate	247.3	223.4	286.26	223.55	42	50.707	[7]
			FE = 0.903		FE = 0.781		FE = 1.207	
500 mg, Intramuscular	Severe	996.45	1087.2	1068.3	1234.5	75	75.815	[7]
			FE = 1.091		FE = 1.156		FE = 1.011	
500 mg, Intramuscular	Severe	845.1	979.82	1232.6	1174.4	59.181	56.145	[53]
			FE = 1.159		FE = 0.953		FE = 0.949	
2000 mg, IV infusion, 1 h	Severe	3007.1	3082.8	3007.1	5563.9	200.71	192.77	[43]
			FE = 1.025		FE = 1.850		FE = 0.960	
500 mg, IM	End-stage	628	704.63	1681.6	2123.1	80	74.004	[7]
			FE = 1.122		FE = 1.263		FE = 0.925	
500 mg, IM	End-stage	1475.6	1434.2	3785.2	4886.7	76	75.261	[7]
			FE = 0.972		FE = 1.291		FE = 0.990	
AFE			1.042		1.172		1.003	

**Table 6:** FE and AFE after IV infusion in healthy pediatrics.

Dose and Route	Age	AUC_0-t_ (µg·h/mL)		AUC_0-∞_ (µg·h/mL)		C_max_ (µg/mL)		Reference
		Observed	Predicted	Observed	Predicted	Observed	Predicted	
25 mg/kg, IV infusion 30 min	3 YO	340.68	347.64	371.43	368.09	240	196.45	[19]
			FE = 1.020		FE = 0.991		FE = 0.819	
25 mg/kg, IV infusion 30 min	4 YO	364.14	448.06	416.25	513.97	238.2	209.21	[19]
			FE = 1.230		FE = 1.235		FE = 0.878	
25 mg/kg, IV infusion 30 min	6 YO	419.74	439.11	599.9	513.88	220	201.24	[19]
			FE = 1.046		FE = 0.857		FE = 0.915	
25 mg/kg, IV infusion 30 min	6 YO	318.19	342.24	396.51	383.86	192.13	187.97	[19]
			FE = 1.076		FE = 0.968		FE = 0.978	
25 mg/kg, IV infusion 30 min	9 YO	345.65	382.19	388.81	422.24	219	200.3	[19]
			FE = 1.106		FE = 1.086		FE = 0.915	
1000 mg, IV infusion 30 min	10–12 YO	617.43	508.83	649.57	514.01	269.46	251.96	[5]
			FE = 0.824		FE = 0.791		FE = 0.935	
2000 mg, IV infusion 30 min	10–12 YO	834.99	885.94	880.56	928.35	344.21	327.96	[5]
			FE = 1.061		FE = 1.054		FE = 0.953	
25 mg/kg, IV infusion 30 min	12 YO	370.41	365.43	415.35	407.81	216.05	188.76	[19]
			FE = 0.987		FE = 0.982		FE = 0.874	
AFE			1.038		0.987		0.907	

**Table 7:** Effects of RI on pharmacokinetic profile.

Dose	CKD Stage	AUC_0-t_ (µg·h/mL)		AUC_0-∞_ (µg·h/mL)		C_max_ (µg/mL)		Dose Adjustment
		Predicted	Elevation Fold	Predicted	Elevation Fold	Predicted	Elevation Fold	
1000 mg (27 mg/kg)	Healthy	515.04	1	515.05	1	251.96	1	-
1000 mg	Mild	577.06	1.1204	577.06	1.12042	253.9	1.0077	No dose adjustment required
1000 mg	Moderate	906.27	1.7596	906.27	1.75961	260.7	1.0347	43% reduction in standard dose
1000 mg	Severe	2566.5	4.9831	2568.2	4.9864	256.32	1.0162	~80% reduction in standard dose
1000 mg	End-stage	4726.1	9.1761	4854.7	9.4257	242.09	0.96082	~90% reduction in standard dose

## Data Availability

The original contributions presented in this study are included in the article/Supplementary Materials. Further inquiries can be directed to the corresponding author(s).

## Supplementary Materials

The following supporting information can be downloaded at: https://www.mdpi.com/article/10.3390/pharmaceutics18101260/s1, Table S1: Alternative Physicochemical Parameter Values Obtained from PubChem and ADMET Predictor^TM^; Table S2: Clinical data used for the development and verification of models for Cefazolin.
