# Supplementary material for: Dose Optimization of Cefazolin for Pediatric Patients with Renal Impairment Using Physiologically Based Pharmacokinetic Modeling (PBPK) in GastroPlus
Source: Pharmaceutics. 2026 Oct 5;18(10):1260. doi: 10.3390/pharmaceutics18101260 (PMC13708336; doi:10.3390/pharmaceutics18101260)
Supplement: Supplementary file 1 [file pharmaceutics-18-01260-s001.zip › pharmaceutics-4547081-supplementary.pdf]

Supplementary Table S1. Alternative Physicochemical Parameter Values Obtained from PubChem and ADMET Predictor™

Parameter	Value	Reference
Physiochemical properties:		
Molecular weight	454.51	ADMET predictor
Log P	-0.58	Pubchem
Log D	3.06 @ pH=6.5	ADMET predictor
Acid pKa (carboxylic acid)	3.6	Pubchem
Blood/plasma ratio (Rbp)	0.69	ADMET predictor

Supplementary Table S2 Clinical data used for the development and verification of models for Cefazolin

Dose	Route of administration	Population	No. of subjects	Reference
**Model Development**				
1g	IV infusion	Healthy Adults	20	[1]
1g	IV bolus	Healthy Adults	19	[2]
**Model Validation**				
2g	IV infusion	Healthy Adults	7	[3]
1g<br>2g	IV infusion<br>IV infusion	Healthy Adults<br>Severe CKD	6<br>1	[4]<br>[4]
2g/3g/4g<br>Single dose	IV infusion	Healthy Adults	7	[5]
4g q12h	IV infusion	Healthy Adults	7	[5]
0.5 g	IV bolus	Healthy Adults<br>(Pregnant, including after pregnancy data)	12	[6]
1g	IV bolus	Healthy Adults	12	[7]
1g	IV bolus	Healthy Adults	13	[8]
0.5g<br>0.5g	IM<br>IM	Healthy Adults<br>Moderate/severe/CKD	5<br>9	[9]<br>[9]
0.5g	IM	Healthy Adults	6	[10]
3g	IV infusion	Healthy Adults	4	[11]
4g	IV infusion	Healthy Adults	25	[12]
1g<br>2g<br>2g	IV infusion<br>IV infusion<br>IV infusion	Healthy Pediatrics<br>Healthy Pediatrics<br>Healthy Adults	12<br>12	[13]<br>[13]<br>[13]
25mg/kg	IV infusion	Healthy Pediatrics	6	[14]
0.5g	IM	Severe CKD	1	[15]

References:

1. Rattie ES, Ravin LJ. Pharmacokinetic interpretation of blood levels and urinary excretion data for cefazolin and cephalothin after intravenous and intramuscular administration in humans. Antimicrob Agents Chemother. 1975;7(5):606–13. doi:10.1128/AAC.7.5.606 PubMed PMID: 1147591.

2. Singhvi SM, Heald AF, Schreiber EC. Pharmacokinetics of cephalosporin antibiotics: protein-binding considerations. Chemotherapy. 1978;24(3):121–33. doi:10.1159/000237771 PubMed PMID: 657874.

3. Lee FH, Pfeffer M, van Harken DR, Smyth RD, Hottendorf GH. Comparative pharmacokinetics of ceforanide (BL-S786R) and cefazolin in laboratory animals and humans. Antimicrob Agents Chemother. 1980;17(2):188–92. doi:10.1128/AAC.17.2.188 PubMed PMID: 7387141.

4. Bhalodi AA, Housman ST, Shepard A, Nugent J, Nicolau DP. Tissue pharmacokinetics of cefazolin in patients with lower limb infections. Antimicrob Agents Chemother. 2013 Nov;57(11):5679–83. doi:10.1128/AAC.01348-13 PubMed PMID: 24041887.

5. Smyth RD, Pfeffer M, Glick A, Van Harken DR, Hottendorf GH. Clinical pharmacokinetics and safety of high doses of ceforanide (BL-S786R) and cefazolin. Antimicrob Agents Chemother. 1979;16(5):615–21. doi:10.1128/AAC.16.5.615 PubMed PMID: 526003.

6. Philipson A, Stiernstedt G, Ehrnebo M. Comparison of the Pharmacokinetics of Cephradine and Cefazolin in Pregnant and Non-Pregnant Women. Clin Pharmacokinet. 1987 Feb;12(2):136–44. doi:10.2165/00003088-198712020-00004

7. Lode H, Gebert S, Hendrischk A. Comparative pharmacokinetics and clinical experience with a new cephalosporin-derivative: cefazolin. Chemotherapy. 1975;21(1):19–32. doi:10.1159/000221840 PubMed PMID: 1097207.

8. Shimada J, Ueda Y. Moxalactam--absorption, excretion, distribution, and metabolism. Rev Infect Dis. 1982;4 Suppl. doi:10.1093/CLINIDS/4.SUPPLEMENT_3.S569 PubMed PMID: 6218568.

9. Rein MF, Westervelt FB, Sande MA. Pharmacodynamics of cefazolin in the presence of normal and impaired renal function. Antimicrob Agents Chemother. 1973 Sep;4(3):366–71. doi:10.1128/AAC.4.3.366 PubMed PMID: 4758839.

10. Kirby WMM, Regamey C. Pharmacokinetics of cefazolin compared with four other cephalosporins. J Infect Dis. 1973;128:S341–6. doi:10.1093/INFDIS/128.SUPPLEMENT_2.S341 PubMed PMID: 4744015.

11. Yamasaku, F.; Kawashima, S.; Takeda, H.; Niwayama, M.; Iwanaga, M.; Tsuchiya, T.; Kinoshita, Y. Pharmacokinetic Study of High-Dose Intravenous Infusion of Cefazolin and Cephalothin. Chemotherapy 1976, 24, 456–464. https://doi.org/10.11250/CHEMOTHERAPY1953.24.456.

12. Adam D, Pätzold J. [Serum concentrations and kinetics after intravenous infusion of four grams of cefazolin (author's transl)]. Infection. 1977 Dec;5(4):228–31. doi:10.1007/BF01640786 PubMed PMID: 598929.

13. Schmitz ML, Blumer JL, Cetnarowski W, Rubino CM. Determination of appropriate weight-based cutoffs for empiric cefazolin dosing using data from a phase 1 pharmacokinetics and safety study of cefazolin administered for surgical prophylaxis in pediatric patients aged 10 to 12 years. Antimicrob Agents Chemother. 2015 Jul;59(7):4173–80. doi:10.1128/AAC.00082-15 PubMed PMID: 25941220.

14. Koshida R, Nakashima E, Ichimura F, Nakano O, Watanabe R, Taniguchi N, et al. Comparative distribution kinetics of cefazolin and tobramycin in children. J Pharmacobiodyn. 1987 Sep;10(9):436–42. doi:10.1248/bpb1978.10.436 PubMed PMID: 3437384.

15. Bergan T, Brodwall EK, ørjavik O. Pharmacokinetics of cefazolin patients with normal and impaired renal function. J Antimicrob Chemother. 1977 Sep;3(5):435–43. doi:10.1093/JAC/3.5.435 PubMed PMID: 903324.
